# Dissecting the transcriptional regulatory networks of promoter-associated noncoding RNAs in development and cancer

**DOI:** 10.1186/s13046-020-01552-8

**Published:** 2020-03-17

**Authors:** Lidia Chellini, Valentina Frezza, Maria Paola Paronetto

**Affiliations:** 1 Laboratory of Molecular and Cellular Neurobiology, IRCCS Santa Lucia Foundation, 00143 Rome, Italy; 2 Department of Movement, Human and Health Sciences, University of Rome “Foro Italico”, Piazza Lauro de Bosis 6, 00135 Rome, Italy

**Keywords:** Promoter-associated noncoding RNA (pancRNA), Chromatin remodeling, Epigenome, Development, Cancer

## Abstract

In-depth analysis of global RNA sequencing has enabled a comprehensive overview of cellular transcriptomes and revealed the pervasive transcription of divergent RNAs from promoter regions across eukaryotic genomes. These studies disclosed that genomes encode a vast repertoire of RNAs beyond the well-known protein-coding messenger RNAs. Furthermore, they have provided novel insights into the regulation of eukaryotic epigenomes, and transcriptomes, including the identification of novel classes of noncoding transcripts, such as the promoter-associated noncoding RNAs (pancRNAs).

PancRNAs are defined as transcripts transcribed within few hundred bases from the transcription start sites (TSSs) of protein-coding or non-coding genes. Unlike the long *trans*-acting ncRNAs that regulate expression of target genes located in different chromosomal domains and displaying their function both in the nucleus and in the cytoplasm, the pancRNAs operate as *cis*-acting elements in the transcriptional regulation of neighboring genes. PancRNAs are very recently emerging as key players in the epigenetic regulation of gene expression programs in development and diseases.

Herein, we review the complex epigenetic network driven by pancRNAs in eukaryotic cells, their impact on physiological and pathological states, which render them promising targets for novel therapeutic strategies.

## Background

Transcription initiation is a tightly regulated process that involves remodeling of nucleosome organization and recruitment of transcription factors and the RNA Polymerase II (RNAPII) on the transcription units. Recent advances based on RNA sequencing efforts documented several classes of long and short noncoding RNAs transcribed in the promoter regions proximal the transcription start sites (TSS) of most annotated genes, bona fide involved in the regulation of transcription initiation [1]. Among them, the promoter-associated noncoding RNAs (named interchangeably pancRNAs or pncRNAs) have now been described in all eukaryotic species, from yeast to human [1–9]. PancRNAs are defined as transcripts transcribed within a few hundred bases from the TSSs of protein-coding or non-coding genes. Unlike the long *trans*-acting ncRNAs that regulate expression of target genes located in different chromosomal domains or even in different chromosomes, the pancRNAs by definition operate as *cis*-acting elements in the transcriptional regulation of neighboring genes [10]. Notably, these noncoding transcripts often show tissue specificity, suggesting regulated expression and functional involvement in biological processes [10].

Here we are reviewing current knowledge about *cis*-acting ncRNAs transcribed from promoter regions of protein-coding genes and their impact on chromatin signature and gene expression programs, in both physiological and pathological conditions.

### Mechanisms of transcriptional regulation by pancRNAs

The first evidence documenting the presence of noncoding transcripts arising from regions located upstream the TSS came from high throughput experiments where *hRrp40*, a *core* component of the human 3′ to 5′ exoribonucleolytic exosome and one of the major RNA degradation complexes, was depleted [11]. In these studies, orientation-specific RT-qPCR performed on RNA from either *hRrp40* depleted- or control cells demonstrated that both sense and antisense transcripts were detectable upstream the TSS, and the transcribed region was characterized by markers of active transcription, such as RNA polymerase II (RNAPII) and acetylated histone 3 (H3K9ac) peaks [11]. Genome-wide and global nuclear run on sequencing confirmed the presence of a population of low-copy small RNAs transcribed from promoter regions of the mammalian genome, shorter than 200 nucleotides, and produced by non-overlapping bidirectional transcription sites [2, 3, 12]. Promoter regions are intrinsically bidirectional [13]. Bidirectionality is achieved though the generation of nucleosome depleted regions via recruitment of nucleosome remodeling complexes that stimulate transcription in both directions [14]. However, sense and antisense divergent transcription rates do not correlate [13] and directionality of transcription units is controlled by a number of regulators, including the Chromatin Assembly Factor I (CAF-I) and factors that promote H3K56 acetylation [15], whereas antisense transcription positively correlates with H4 acetylation [13]. A fascinating hypothesis is that these bidirectional transcripts may act as a RNA reservoir forged by evolutionary pressures to support physiological functions [16, 17].

**Fig. 1:**
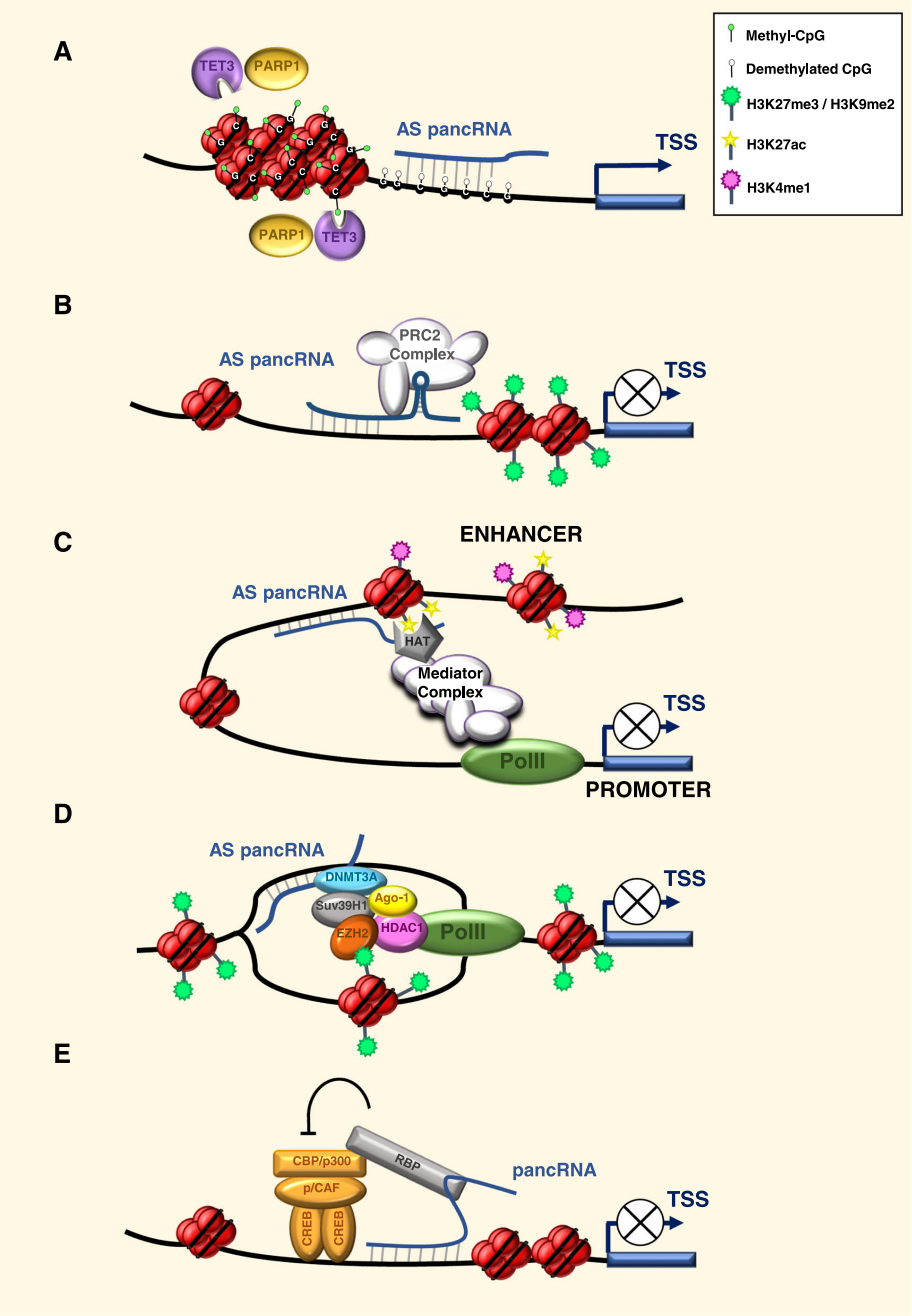
Hypothetical models to explain sense and antisense pancRNA (AS pancRNA) activities. **a** pancRNAs act in concert with PARP1 and TET3 factors to establish the hypomethylated state of CpG islands within the promoter, thus activating in *cis* the transcription of the host gene. **b** AS pancRNAs recruit Polycomb repressive complex PRC2, catalyzing the trimethylation of histone H3 at K27 and the chromatin packaging. **c** AS pancRNAs can bind histone acetylases and methylases that open the chromatin, allowing the engagement of the Mediator Complex and the chromatin looping. **d** Antisense pancRNAs tight on the promoter an epigenetic silencing complex formed by Ago-1, DNMT3a, EZH2, Suv39H1 and HDAC1, which causes trimethylation of histone H3 at K27, heterochromatin formation and repression of transcription. **e** pancRNAs can bind RNA binding proteins (including FUS/TLS and Sam68), thus promoting inhibition of the histone acetyltransferase activity of the p300/CREB binding protein (CBP)-associated factor (PCAF) and resulting in the suppression of transcription

How these noncoding transcripts, both sense and antisense, are involved in the regulation of their host genes is still under investigation, and multiple mechanisms have been proposed, leading either to the activation or repression of the host genes. One hypothesis is that pancRNAs act as mediators of sequence-specific epigenetic changes. To this regard, one proposed molecular mechanism involves the control of CpG (de) methylation, as described for the *Sphk1* gene and its *Khps1* pancRNA variants [18]. A similar mechanism was observed in mouse embryos, where expression of bidirectional pancRNAs was strongly associated with the upregulation of their host genes during the zygotic genome activation at the 2-cell stage accompanied by sustained DNA demethylation [19], potentially driven by *trans*-acting factors, such as TET (**T**en **E**leven-**T**ranslocation, Methylcytosine Dioxygenase) proteins, to achieve a sequence-specific DNA demethylation (Fig. 1a).

An alternative, but not mutually exclusive possibility, is that pancRNAs display a more general function, facilitating rapid activation or repression of the downstream gene by altering chromatin structure or by recruiting transcription regulators. Short RNAs originating within 700 base pairs (bp) upstream the TSS of genes targeted by Polycomb were identified in primary T cells and embryonic stem cells [20]. These RNAs form a stem-loop structure interacting with SUZ12, a component of the Polycomb repressive complex 2 (PRC2). Recruitment of the complex achieves histone H3 Lys27 trimethylation (H3K27me3) thus causing repression of the gene [20, 21] (Fig. 1b).

Another functional model derives from initial work performed in *S. pombe* [22] and confirmed in humans [23], where the pancRNAs variants are involved in RNA-mediated contribution to the recruitment of transcription factors to enhancers and promoters. This model would suggest that bidirectional transcription of active enhancers and promoters evolved to facilitate trapping of transcription factors at specific regulatory elements, producing a positive feedback loop that contribute to the establishment of gene expression programs (Fig. 1c).

In a further model, the RNAPII reading through the promoter allows transcription of low copy pancRNAs targeted by antisense RNAs. The pancRNAs and antisense RNA form a complex that associate with the local chromatin architecture through a chromatin remodeling complex presumably containing histone methyltransferase DNMT3A. Remarkably, the antisense RNAs-pancRNAs form RNA:RNA hybrids that create docking sites for the recruitment of gene silencing complexes [24, 25]. In particular, in this model pancRNAs containing an extended 5′ UTR are recognized by endogenous antisense RNAs during RNAPII-mediated transcription of the RNA-targeted promoter. The antisense strand guides a silencing complex composed by DNMT3A, Ago-1, HDAC-1, and/or EZH2 to the targeted promoter (Fig. 1d). Next, the antisense RNA-targeted promoter may result in heterochromatinization of the local siRNA-targeted genomic region, exhibiting a silent shape, marked by both histone H3 lysine-9 di-methylation (H3K9me2) and histone H3 lysine-27 tri-methylation (H3K27me3) [24].

In addition to RNAPII, pancRNAs transcribed by RNAPI in the nucleolus have been shown to take part to regulatory networks controlling the epigenetic state of chromatin of ribosomal genes. These pancRNAs form a DNA:RNA triplex recognized by the DNA methyltransferase DNMT3B to achieve de novo CpG methylation of rRNA genes (rDNA) [26]. The formation of DNA-RNA hybrid structures has been proposed to explain the unmethylated status of most human CpG islands in promoter regions. Unlike the previous model, unmethylated CpG promoters in the human genome show strand asymmetry in the distribution of guanine and cytosines, a property known as “GC skew”. This property confers the ability to form stable, three-stranded structures called R-loops upon RNA transcription. R-loop formation at promoters can prevent binding of DNMT3B and, therefore, DNA methylation [27].

Another mechanism proposed for pancRNA function has been illustrated for the cyclin D1 gene (*CCND1*) [28, 29]. Wang and colleagues described DNA damage-driven pancRNAs transcribed in HeLa cells from the region upstream the TSS of *CCND1* gene. The authors showed that the *pancCCND1_D* was able to recruit the RNA binding protein FUS and CBP/ p300, thus inhibiting the histone acetyl transferase (HAT) activity, resulting in *CCND1* repression (Fig. 1e). Similarly, Palombo and colleagues described a cognate pancRNA transcribed from the same region, the *pancCCND1_B*, expressed in Ewing sarcoma and actively recruiting a multimolecular complex composed by the RNA binding protein Sam68 and the RNA/DNA helicase DHX9 to inhibit *CCND1* transcription [29]. These studies highlight the possibility that multiple transcripts driven by the same promoter can synergistically act in *cis* to achieve activation or repression of the host gene, or even play antagonistic functions.

Although initial models focused on pancRNAs involved in the epigenetic silencing of nearby genes, later on genome-wide efforts demonstrated a positive correlation between the expression of pancRNAs and the neighboring protein-coding genes [30, 31]. The emerging scenario indicates both enhancing and repressive activities exerted by pancRNAs on their host target genes depending on the intrinsic characteristics of each pancRNA and on the regulatory features imposed by the local chromatin conformation.

### pancRNAs: regulators of transcription in developmental processes

In the past few years, pancRNAs have been shown to cover crucial roles in the guidance of cell differentiation [18, 19, 32, 33]. Transcriptome profiling of human and murine embryonic stem cells revealed that more than 60% of the lncRNA derive from divergent transcription at the promoters of protein-coding genes. Analysis of the distribution of these divergent pancRNAs revealed that their position in the genome is not casual, but it is correlated with protein-coding genes enriched in functions linked to developmental processes [34]. In fact, the pancRNA/mRNA gene pairs undergo coordinated transcriptional changes during the differentiation of the human embryonic stem cells toward endoderm [23]. Furthermore, transcriptome signatures revealed that bidirectional pancRNAs display different expressions pattern in human fetal and adult heart, due to a fine-tuned orchestration of specific epigenetic modifications, suggesting that pancRNAs which show a mutual expression with their neighboring genes, may contribute to the fetal heart development [35].

It is well known that promoters with a high CpG content can undergo bidirectional transcription and that divergent lncRNAs are capable to positively regulate in *cis* the transcription of the nearby coding genes, by contributing to their epigenetic setting [33]. As mentioned above, one of the first evidence documenting the modulation of gene expression depending on the bidirectional activity and the methylation status of the promoter was provided for the sphingosine kinase 1 (*Sphk1*) gene, in primary cells from rat fetuses [18]. Sphingosine kinase 1 is involved in the differentiation of fibroblast and keratinocytes, as well as in vascular and neural development. The *Sphk1* CpG island contains a regulatory element, indicated as tissue-dependent differentially methylated region (T-DMR), involved in the regulation of *Sphk1* expression. Transcription of the antisense pancRNA *Khps1* from the promoter induces DNA demethylation at CG sites, specifically in the T-DMR of *Sphk1*, thus inducing de-repression of the gene [18]. A further proof that pancRNAs can induce the demethylation of CpG islands upstream the partnered ORF, came later from a study conducted in normal rat kidney epithelial cells and undifferentiated PC12 rat cell line [32]. This function of the pancRNAs has been demonstrated as essential for the zygotic gene activation and the acquisition of pluripotency in mouse early embryos but also for meso- and endodermal differentiation of pluripotent murine embryonic stem cells [19, 36]. Initially, in mouse preimplantation embryos, the pancRNA *pancIl17d* turned out to be responsible for triggering the expression of the interleukin 17d, indispensable for successful formation of the blastocyst. Although the expression of this pancRNA was associated with an increased DNA demethylation at the promoter, the molecular mechanism underlying this regulation was not further characterized. The epigenetic modifications associated to the zygotic gene activation was assumed to be due to the enrollment of PARP and TET3 demethylation factors on chromatin by pancRNAs or to be possibly only an indirect upshot [19, 37].

In addition, it has been proposed that pancRNAs might bind epigenetic factors, promoting the fold of the chromatin into a transcriptionally competent ternary structure, named R-Loop and composed by the nascent RNA annealed with its complementary ssDNA and the non-template ssDNA. Another possibility was that pancRNAs may perhaps stabilize the active state of chromatin through the association with small dsRNAs, which in turn, could cover the catalytic site of methyl transferases [38].

A subsequent investigation carried out in murine ESC, underlined the requirement of *Evx1as* lncRNA for the transcriptional activation of the neighboring protein-coding gene *EVX1*, essential for mesoderm differentiation [36]. In this case, the authors successfully demonstrated that the pancRNA binds an enhancer site on its *locus* and enables the recruitment of the transcription co-activator Mediator on the promoter [36]. Mediator thus coordinates the enhancer–promoter gene loop required for the assembly of the transcription machinery (Fig. 1c) [36].

Coupled expression of pancRNAs and the relative mRNAs in differentiating cells is often supported by the enrichment of histone acetylation and methylation in the promoter [34]. The terminal differentiation of PC12 cells is cAMP-dependent and relies on the fine-tuned expression of several pancRNAs deriving from bidirectional promoters enriched in cAMP response elements [39]. Among them, *pancNusap1* positively regulates the expression of the spindle-associate gene *Nusap1*, and this effect is accompanied by an increased acetylation of H3 lysine 9 and 27 at the promoter [39]. The stimulation with cAMP induces a CRE-mediated concerted repression of *pancNusap1* and *Nusap1*, resulting in the inhibition of the M-phase and the irreversible cell cycle withdrawal [39]. Similarly, *Uph1* pancRNA regulates in *cis* the transcription of its paired coding gene *Hand2* by enhancing the histone modifications H3K4me1 and H3K27ac in the enhancer [40]. The function of *Uhp* is critical for heart development in mice embryos, as demonstrated by the severe cardiac defects of *Uph−/−* mice, leading to embryonic lethality at stage E10.5 [40]. A possible mechanism explaining the increased histone acetylation comes from recent data documenting that during myogenic differentiation, the pancRNA *Myoparr* binds the myogenin shared promoter and physically interacts with the transcriptional co-activator DDX17 and the histone acetylase PCAF. This association leads to increased H3K27 acetylation of the promoter and the subsequent recruitment of the transcription factor MYOD1 [41]. This also allows the maximum occupancy of the RNAPII on the *Myogenin locus*, contributing to the exit of myoblasts from cell cycle and promoting differentiation [41].

Remarkably, recruitment of the Mediator complex on the E*VX1* promoter is associated to the H3K27ac and H3K4me1 histone modifications [36], where the histone acetylases recruited by the antisense pancRNA empower the access of Mediator. Some antisense poly(A) + pancRNAs from bidirectional promoters can also antagonize the transcription of their relative sense mRNAs. As an example, the pancRNA *hTAPAS* represses the gene encoding human telomerase reverse transcriptase (hTERT). In the germline and human embryonic stem cell line *hTAPAS* is fundamental for keeping low the concentration of the TERT protein, guarantying telomere length homeostasis and proliferative potential in the cells [42]. It is interesting to note that some antisense pancRNAs do not regulate the expression of the nearby *locus*, but can modulate in *trans* the transcription of other genes, by functionally interacting with the protein encoded from their cognate sense transcript, as for the pancRNA *Six3OS*, that modulates the activity of the transcriptional factor SIX3, by linking chromatin-modifying enzyme complexes on the SIX3 target genes [43]. This mechanism seems required for cell specification in the postnatal retina and the correct eye development in mice [43]. Interestingly, in the crustacean *Daphnia magna* it has been recently characterized the sense capped but non-polyadenylated pancRNA *DAPALR*, that can activate in *trans* the transcription of the downstream locus *dsx1*, relevant for sex determination during embryogenesis [6]. The molecular mechanism underlying the action of *DAPALR* is still unclear, but this discovery denotes that the developmental role of pancRNAs is conserved in all eukaryotes although displaying diversification for directionality of transcription, post-transcriptional modifications and mechanism of action. Interestingly, antisense lncRNAs and sense transcripts tend to have a concerted expression in a tissue-specific fashion in fully developed organs, including cerebral cortex, cerebellum, heart, kidney and liver [33, 34]. This trend is conserved between mammalian species (chimpanzee, macaque, marmoset, mouse and rat), suggesting that pancRNAs participate in the maintenance of tissue homeostasis in adults [33, 34]. To this regard, it has been shown that pancRNAs undergo a strong activation and are involved in the response to oxidative stress in somatic cells [44]. In particular, after exposure to reactive oxygen species, pancRNAs associate with polysomes in the cytoplasm and compete with mRNAs, lowering the translational efficiency and favoring stress adaptation [44].

All these evidences support the idea that pancRNAs display evolutionary conserved functions emblematic both for differentiation and homeostasis in all eukaryotes.

### Cancer-related scaffolding function of pancRNAs

Alterations of pancRNAs have been extensively linked to the changes of gene expression programs occurring in human cancer. PancRNAs affect epigenetic modification of promoters through scaffolding function. It has been shown that non-protein coding transcripts display key roles in cancer biology by impacting the gene expression of important regulatory proteins [45]. In particular, emerging evidences report the ability of ncRNAs to fold into a tertiary structure providing a scaffold for other proteins or regulatory molecules, including RNAs [45].

It is well established the oncogene-like role of pancRNAs in tumorigenesis through the control of critical cell cycle regulators like cyclins [28, 29]. In response to DNA damage such as ionizing irradiation (IR), the *CCND1* pancRNAs are transcribed and negatively regulate cyclin D1 expression [28]. In HeLa cells, pancRNAs transcribed from the promoter of *CCND1* gene associate with and recruit the RNA binding protein FUS/TLS (Fused in sarcoma, Translocated in liposarcoma). The localization of the *pancRNA_CCND1*-FUS complex to the *CCND1* promoter results in the inhibition of the CBP/p300 histone acetyltransferases activity thereby preventing *CCND1* transcription [28]. More recently, a similar repression of the cyclin D1 expression mediated by a protein-RNA complex was described in Ewing sarcoma cells [29]. A deep investigation of the molecular mechanisms underlying this regulation showed that after mitogenic stimuli, such as IGF-1 treatment, the protein partners EWS-FLI1 and DHX9 interact promoting the transcription of target genes, such as *CCND1*. Perturbation of the EWS-FLI1/DHX9 interaction by the small molecule YK-4-279 causes the formation of an alternative inhibitory complex composed by the RNA binding protein Sam68, DHX9, and the *pancRNA_CCND1_B* determining a repressive chromatin environment responsible of cyclin D1 transcriptional inhibition. The ability of pancRNAs_*CCDN1* to affect cell proliferation through transcriptional regulation of *CCND1* reveals the contribution of these molecules in the control of cell cycle progression and in the tumorigenic process [29].

A similar mechanism has been documented for the *pancRNA_Ets-1,* displaying a role in the promotion of in vivo*/*in vitro growth and aggressiveness of gastric cancer [46]. *PancRNA_Ets-1* facilitates the physical interaction between the RNA binding protein NONO and the transcriptional factor ERG, resulting in transactivation of ERG and increased cancer-related gene transcription [46]. Moreover, *pancRNA_Ets-1* displays a crucial role in neuroblastoma tumorigenesis, by promoting neuroblastoma cell growth, invasion and metastasis. *PancRNA_Ets-1* binds to the heterogeneous nuclear ribonucleoprotein K (hnRNPK) to facilitate its physical interaction with β-catenin, resulting in reduced β-catenin proteasome-dependent degradation [47]. As a consequence, the enhanced nuclear translocation of β-catenin affects its gene transcriptional program contributing to neuroblastoma progression. Notably, *pancRNA_Ets-1* upregulation in clinical tissues and cell lines of both gastric cancer and neuroblastoma could be exploited as promising markers and potential therapeutic targets.

### RNA-directed transcriptional gene regulation by pancRNAs in cancer

The *pancRNA_FOXCUT* is transcribed upstream of the Forkhead box C1 (*FOXC1*) gene and its expression is positively correlated with *FOXC1* mRNA [48]. The transcription factor FOXC1 is a key regulator of several biological processes and its abnormal expression was found associated to poor survival in various malignant tumors and in the epithelial to mesenchymal transition (EMT) process [24, 49, 50]. Recent studies showed that the FOXCUT–FOXC1 regulatory network is associated with tumorigenesis and cancer progression in esophageal squamous cell carcinoma (*ESCC*) [50], oral squamous cell carcinoma (OSCC) [51] and nasopharyngeal carcinoma (NPC) [52]. *FOXCUT* expression was found enhanced in Basal-Like Breast Cancers (BLBCs) but not in other non-basal like breast cancer subtypes, suggesting that *FOXCUT* may function as specific biomarker in BLBCs [53]. Notably, in all these different tumor contexts, knockdown of *FOXCUT* reduced the levels of *FOXC1* mRNA, concurring with the observed inhibition of migration, invasion and the metastatic potential of cells.

Similarly, the expression of the *pancRNA_HIF2PUT* (HIF2PUT) was found correlated with the expression of the hypoxia-inducible factor-2α (HIF-2α), in osteosarcoma patients [54, 55]. HIF-2α has been associated with stem-like properties in stem cells and in cancer stem cells of different types of tumor [46, 56, 57]. Moreover, *HIF2PUT* knockdown is able to induce enhanced proliferation, migration and self-renewal of MG63 cells, suggesting that *HIF2PUT* may suppress the properties of cancer stem cells in osteosarcoma by regulating the expression of HIF-2α [54]. In colorectal cancer specimens, the expression of *HIF2PUT* positively correlates with HIF-2α expression and *HIF2PUT* knockdown impairs the stem cell like properties of colorectal cancer stem cells by regulating HIF-2α expression [58].

In different prostate cancer cell lines and tumors, Pisignano and colleagues showed that the gene encoding E-cadherin (*CDH1*), known as an epithelial cell differentiation marker and a tumor suppressor, is epigenetically silenced contributing to the acquisition of key essential properties in the tumor development and progression. They found that a specific miRNA (isomiR-4534) guides the Argonaute1 (Ago1) binding to a precise site on the *pancRNA_CDH1*. In turn, Ago1 recruits the histone methyltransferase SUV39H1 to repress the promoter activity with the consequent transcriptional suppression. Importantly, a cancer-associated single nucleotide polymorphism (SNP, rs16260) facilitates the accessibility of the miRNA-Ago1 complex to the *pancRNA_CDH1*, increasing *CDH1* gene silencing [59].

As mentioned above, pancRNAs can also be involved in the formation of a RNA:DNA hybrid (named R-loop) to affect gene expression. This kind of mechanism was found in colon cancer cells and in two different breast cancer cell lines, MCF7 and MCF10, where the *pancRNA_VIM*, an antisense noncoding transcript on Vimentin RNA (deposited as *VIM-AS1*), supports an open chromatin state by participating to the formation of R-loop. This structure facilitates the accessibility of Vimentin gene to the transcriptional machinery and enhances the NF-Kβ binding to Vimentin promoter, thus resulting in increased gene transcription [60].

Beside the possibility to form a R-loop, pancRNAs may be involved in the formation of a DNA-RNA triplex. Recently, it has been reported that the expression of the proto-oncogene SPHK1, involved in the control of apoptosis, requires a pancRNA, termed *Khps1*, transcribed in antisense orientation to *SPHK1*. *Khps1* is bound to a homopurine stretch (TFR2), forming a DNA-RNA duplex able to interact with p300/CBP, thus establishing an open chromatin structure that facilitates the E2F1-mediated *SPHK1* transcription [61].

Collectively, these studies (listed in Table 1) suggest a role of pancRNAs in carcinogenesis. The discovery of the precise mechanisms through which these molecules may modulate transcription of cancer-related genes could provide a deeper understanding of the complex integrated network that supports the tumor phenotype.

**Table 1:** List of pancRNAs identified in cancer

Name	Cancer Type	Proposed Mechanism	Downstream Effects	Ref.
*pancRNAs_CCDN1*	Cervical cancer	Inhibition of CBP/p300 HAT activity by *pancRNA_CCDN1*-FUS complex	Repression of Cyclin D1 expression	[28]
	Ewing sarcoma	The *pancRNA_CCND1_B*/Sam68/DHX9 complex determines a repressive chromatin environment		[29]
*pancRNA_Ets-1*	Gastric cancer	NONO-*pancRNA_Ets-1* complex interacts with ERG	Activation of ERG-related gene transcription	[46]
	Neuroblastoma	Interaction between *pancRNA_Ets-1*/hnRNPK complex and β-catenin	Enhanced β-catenin transcriptional activity	[47]
*FOXCUT*	Esophageal squamous cancer	Unknown	Positive correlation with FOXC1 expression	[50]
	Oral squamous cell carcinoma			[51]
	Nasopharyngeal carcinoma			[52]
	Basal-like breast cancer			[53]
*HIF2PUT*	Osteosarcoma	Unknown	Positive correlation with HIF-2α expression	[54, 55]
	Colorectal cancer			[58]
*pancRNA_CDH1*	Prostate cancer	IsomiR-4534/Ago-*pancRNA_CDH1* recruits SUV39H1	*CDH1* transcriptional repression	[59]
*pancRNA_VIM*	Colon cancer	R-loop to support an open chromatin state	Enhanced Vimentin transcription	[60]
	Breast cancer			
*Khps1*	Cervical cancer, liver cancer and osteosarcoma	DNA-RNA duplex able to interact with p300/CBP	Enhanced E2F1-mediated *SPHK1* transcription	[61]

## Conclusions

Genome-wide transcriptomic analyses have enabled huge advances in the understanding of the intricate transcriptional regulatory network driven by *cis*-acting noncoding transcripts on their target genes, highlighting enhancing and repressive activities exerted by the pancRNAs on target genes [62]. Accumulating evidence shows that changes in the repertoire of pancRNAs finely shape tissue-specific gene expression patterns [34]. Indeed, comparative transcriptome analyses revealed higher diversity of pancRNA- over mRNA-expression profiles, favoring an evolutionary diversification of the transcriptome according to a given species [34]. In parallel, promoter regions of pancRNA-hosting genes exhibit a higher level of sequence conservation than those of pancRNA-lacking genes [34]. Thus, pancRNAs could represent a new layer of species-dependent gene regulation mechanism that allows and contributes to the dynamic evolutionary process and adaptation of a species.

The differential expression of pancRNAs between tumor and benign specimens suggest their potential usage as diagnostic, prognostic or predictive biomarkers. A fascinating question is whether single-nucleotide polymorphisms (SNPs) in the promoter regions affect the function of these promoter-proximal transcripts, by impacting chromatin dynamics and transcription factor recruitment. To this regard, several disease-associated SNPs map to noncoding regions, including promoters [63, 64], but whether pancRNAs are involved in the disease process and the mechanistic link between noncoding SNPs and transcriptional regulation has not been clarified yet, although possible effects of deleting regulatory elements contained within the mutated regions could not be excluded. Furthermore, given the relevance of pancRNAs in developmental processes and in pathological conditions, the potential of the endogenous pancRNA as a therapeutic target could be further evaluated and exploited. For example, forced expression of pancRNAs could be instrumental for artificially manipulating epigenetic modification on specific candidate genes, to accomplish a targeted and functional disruption of aberrant methylation activity, with important implications in transcriptional reprogramming. Conversely, targeted deregulation of pancRNA-based transcriptional network could contribute to epigenetic silencing of tumor suppressor genes and disease progression, thus altering the epigenetic landscape of patients.

Collectively, the reported findings highlight pancRNAs as potential candidates for transcriptional reprogramming. To this regard, the epigenome profile could be re-written by a set of epigenetic modifiers, including pancRNAs. Manipulations of these molecules could be employed in medical purposes.

## Data Availability

Not applicable.

## Abbreviations

Ago1: Argonaute1
BLBCs: basal-like breast cancers
CAF-I: Chromatin Assembly Factor I
CCND1: cyclin D1 gene
EMT: epithelial to mesenchymal transition
ESCC: esophageal squamous cell carcinoma
H3K27me3: histone H3 Lys27 trimethylation
H3K27me3: histone H3 lysine-27 tri-methylation
H3K9ac: acetylated histone 3
H3K9me2: H3 lysine-9 di-methylation
HAT: histone acetyl transferase
hnRNPK: heterogeneous nuclear ribonucleoprotein K
NPC: nasopharyngeal carcinoma
OSCC: oral squamous cell carcinoma
pancRNA: Promoter-associated noncoding RNA;
RNAPII: RNA polymerase II
SNP: single nucleotide polymorphism
Sphk1: the sphingosine kinase 1
TSS: Transcription Start Site
